# Structure-based identification of salicylic acid derivatives as malarial threonyl tRNA-synthetase inhibitors

**DOI:** 10.1371/journal.pone.0296995

**Published:** 2024-04-01

**Authors:** Raitis Bobrovs, Jekaterina Bolsakova, Jhon Alexander Rodriguez Buitrago, Larisa Varaceva, Marija Skvorcova, Iveta Kanepe, Anastasija Rudnickiha, Emilio Parisini, Aigars Jirgensons

**Affiliations:** 1 Latvian Institute of Organic Synthesis, Riga, Latvia; 2 Department of Chemistry “G. Ciamician”, University of Bologna, Bologna, Italy; Kafrelsheikh University Faculty of Pharmacy, EGYPT

## Abstract

Emerging resistance to existing antimalarial drugs drives the search for new antimalarials, and protein translation is a promising pathway to target. Threonyl t-RNA synthetase (ThrRS) is one of the enzymes involved in this pathway, and it has been validated as an anti-malarial drug target. Here, we present 9 structurally diverse low micromolar *Plasmodium falciparum* ThrRS inhibitors that were identified using high-throughput virtual screening (HTVS) and were verified in a FRET enzymatic assay. Salicylic acid-based compound (LE = 0.34) was selected as a most perspective hit and was subjected to hit-to-lead optimisation. A total of 146 hit analogues were synthesised or obtained from commercial vendors and were tested. Structure-activity relationship study was supported by the crystal structure of the complex of a salicylic acid analogue with a close homologue of the plasmodium target, *E*. *coli* ThrRS (*Ec*ThrRS). Despite the availability of structural information, the hit identified via virtual screening remained one of the most potent *Pf*ThrRS inhibitors within this series. However, the compounds presented herein provide novel scaffolds for ThrRS inhibitors, which could serve as starting points for further medicinal chemistry projects targeting ThrRSs or structurally similar enzymes.

## Introduction

Malaria is a life-threatening infectious disease transmitted by mosquitoes and mainly affects people living in tropical and subtropical regions [1]. Annually, the disease claims the lives of around 600 thousand people, mostly children below 5 years old. The disease is caused by plasmodium parasites, the most dangerous species of which is *Plasmodium falciparum*. A broad arsenal of therapeutics is available for preventing and curing malaria; however, drug-resistant strains have been reported to all the clinically used anti-malarial drugs [2]. This alarming spread of resistance motivates the search for new anti-malarial drugs with yet-to-be-exploited modes of action.

**Fig 1:**
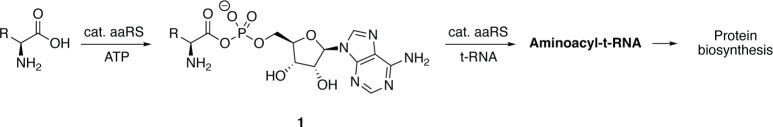
Enzymatic reactions of aminoacyl t-RNA synthetases (aaRS).

Aminoacyl t-RNA synthetases (aaRS) are ligases that conjugate a t-RNA with the cognate amino acid to ensure protein biosynthesis in the ribosome. The enzymatic reaction consists of a two-step process, the first involving the formation of aminoacyladenosine, and the second featuring the transfer of the amino acid from this reactive intermediate to the cognate t-RNA (Fig 1). This group of enzymes have been explored extensively as targets for the development of anti-infective agents, resulting in the marketed drug Mupirocin (IleRS inhibitor) and in many other clinical or preclinical candidates [3–9].

**Fig 2:**
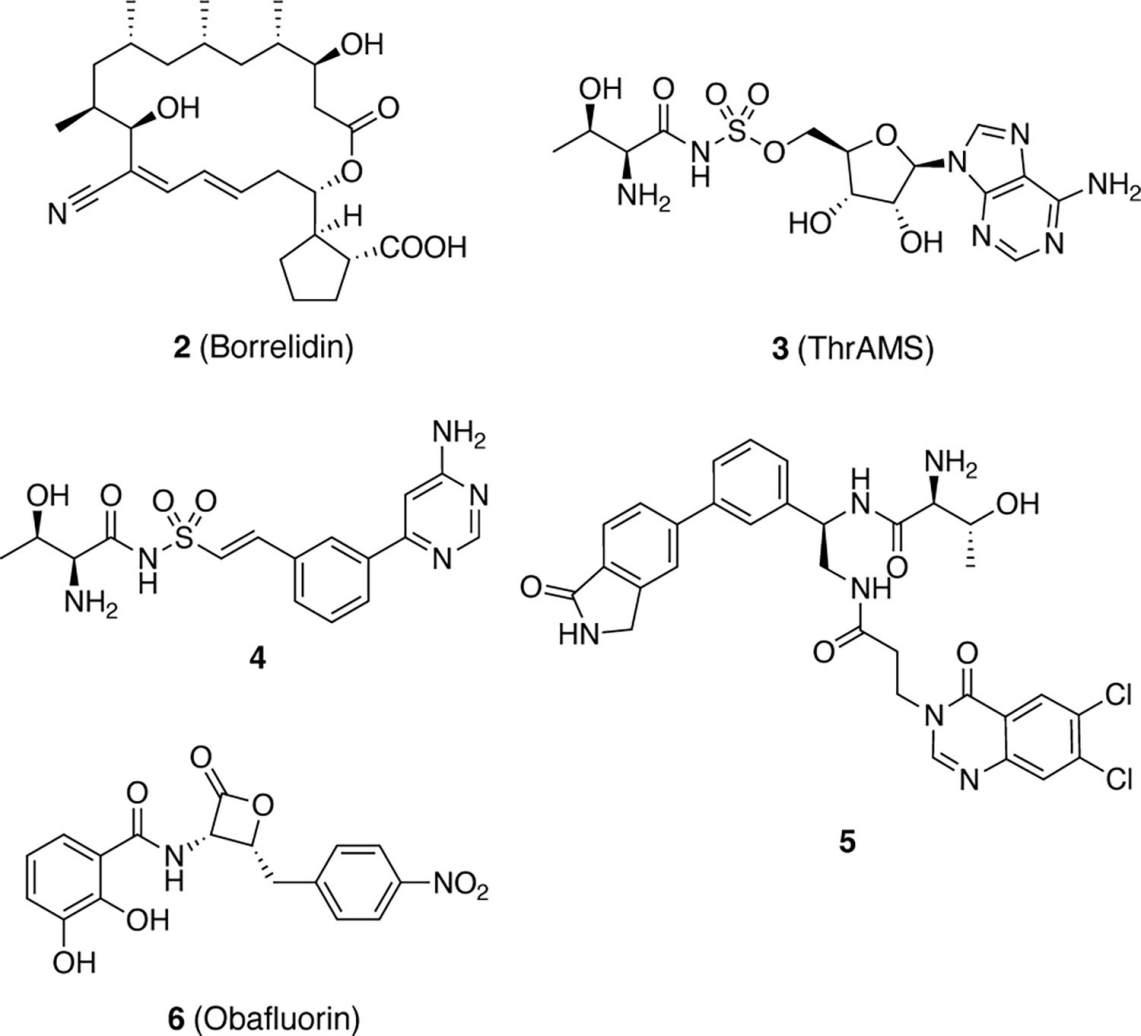
The representative inhibitors of threonyl t-RNA synthetases (ThrRS).

Several aaRS have attracted attention as potential antimalarial targets [10–12]. Among them, threonyl t-RNA synthetase (ThrRS) has been validated together with its inhibitor, the natural product borrelidin (compound **2**, Fig 2) and its analogues, which have shown to possess anti-malarial activity when tested both in infected cell assays and in mice infection models [13–15]. Inhibitors of ThrRS of other organisms are known, e.g. compound **3** (ThrAMS) is a non-cleavable analogue of aminoacylation intermediate **1**, dual site inhibitor **4** [16], triple site inhibitor **5** derived from inhibitors occupying t-RNA binding sub-pocket [17–19] and covalent inhibitor **6** (Obafluorin) [20]. Since known malarial ThrRS inhibitors are still limited to borrelidin and its analogues, as well as aminoacylation intermediate analogues [21], here we applied virtual high throughput screening to identify novel ThrRS inhibitors based on different chemotypes.

## Results and discussion

### Virtual high throughput screening

**Fig 3:**
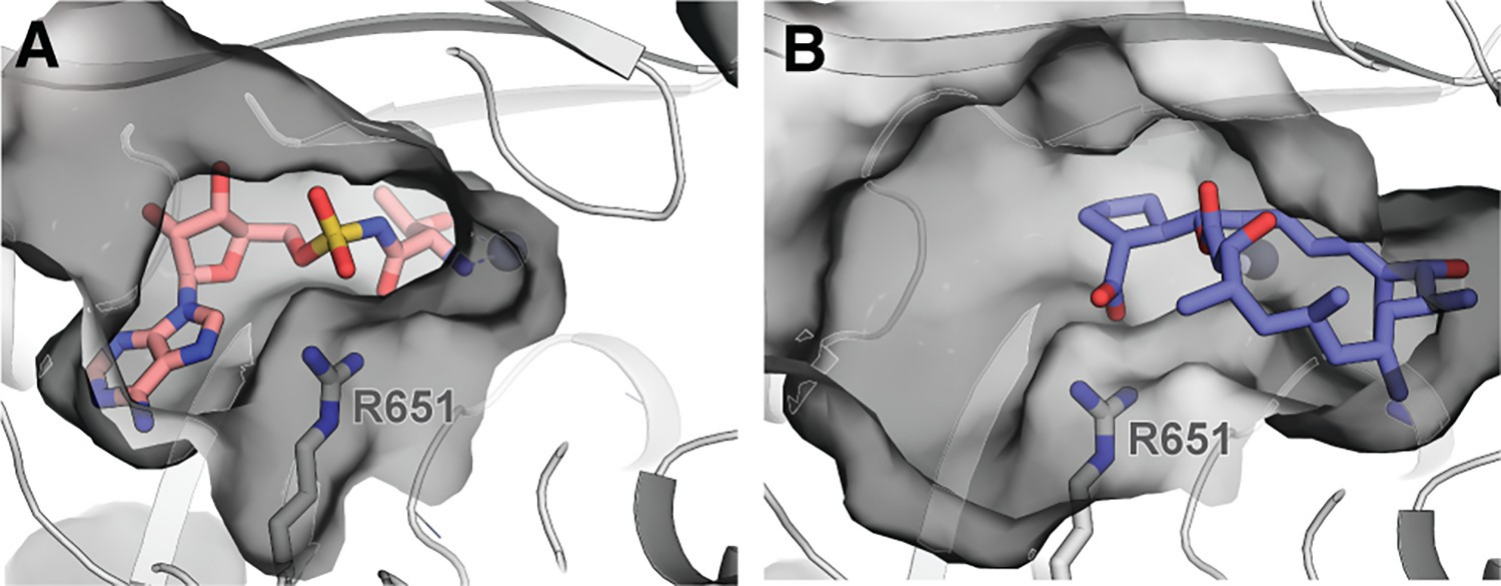
ThrRS binding site in the case of (A) ThrAMS (salmon, PDB ID: 1EVL) and (B) borrelidin (violet, PDB ID: 4P3N) binding. The binding site residue Arg651 side chain is shown as grey sticks. Hydrogens are omitted for clarity. The binding site shape is depicted using surface representation.

Since no crystal structure of *Pf*ThrRS is available, computational studies were initiated by homology modelling. *Pf*ThrRS models suitable for HTVS were created using two bacterial ThrRS structures as templates–one in complex with substrate analogue ThrAMS (PDB ID: 1EVL) [22], and another one with natural product borrelidin (PDB ID: 4P3N) [10]. Each of these inhibitors is known to bind to a slightly different conformation of the ThrRS binding site (see Fig 3); hence, to maximise the possibility of identifying novel scaffolds as ThrRS inhibitors, both conformations were explored.

**Fig 4:**
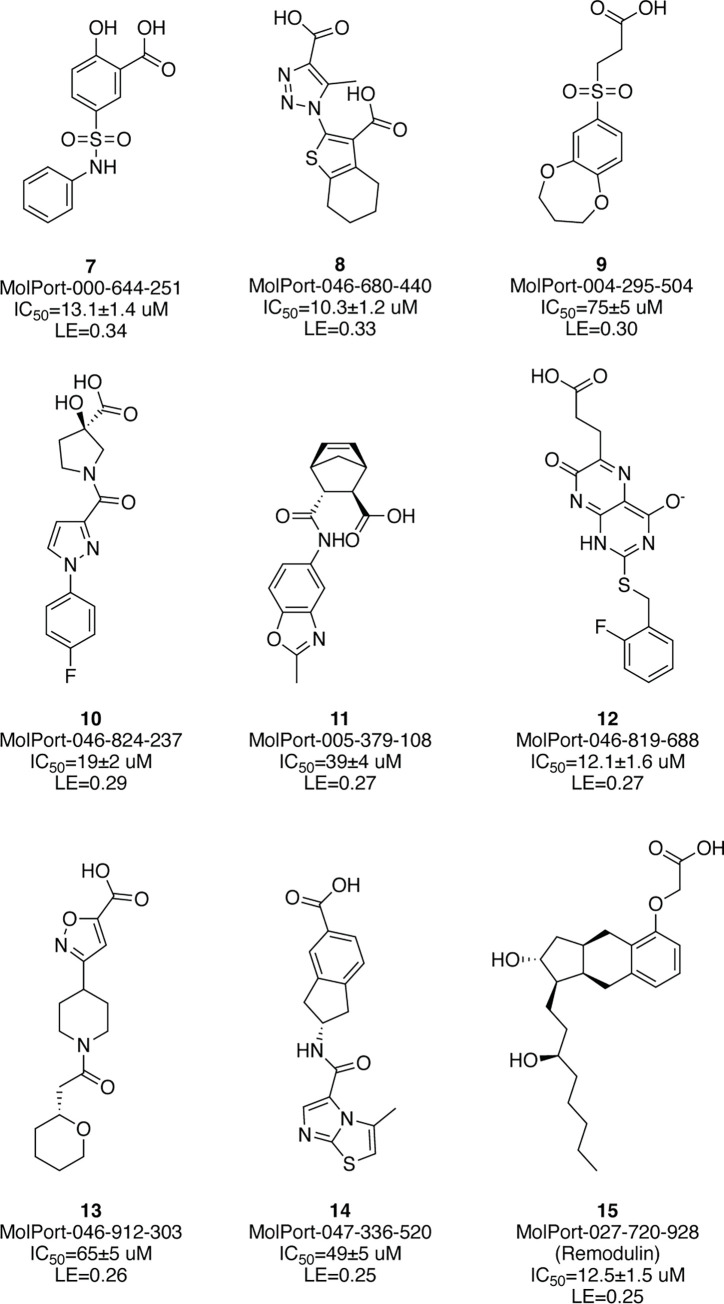
Verified PfThrRS inhibitors arranged according to ligand efficiency (LE). Compound MolPort IDs, IC_50_ values and LE are given under the compound molecular structure.

Potential *Pf*ThrRS inhibitors were identified via high throughput virtual screening (HTVS) of the commercially available library of compounds MolPort (~6.3M comp., 2021) and a collection of open natural products (COCONUT; ~400k comp., 2021) [23] against homology models of the target protein (see Methods section). The top-ranked molecules were inspected for their ability to form hydrogen bonding and stacking interactions similar to those observed in the available ThrRS homologue complex structures (e.g., hydrogen bonding with Arg651, Glu653, Val664 main chain, Gln669, Asp671, Tyr749, Gln677 and/or Gln771; and stacking interactions with Phe667). Molecules featuring internal conformational strain or unsatisfied hydrogen bond donors/acceptors were deprioritised. A total of 58 compounds were purchased from MolPort and tested in an enzymatic assay (see Methods section; 40 compounds expected to bind in a ThrAMS binding site; 18 in a more open ThrRS binding site conformation, similar to that of borrelidin). Of these compounds, 21 compounds showed *Pf*ThrRS inhibition potency with IC_50_ values lower than 80 μM (20 compounds from the aminoacyl binding site subset, and 1 from the borrelidin binding site subset). Some of the most potent hits are shown in Fig 4. All verified *Pf*ThrRS inhibitors are carboxylic acids; this is not surprising, since it was expected that the carboxylic acid group would coordinate the zinc ion in the binding site in a similar fashion as hydroxyl and amino groups of threonine. Further away from the carboxylic acid group that coordinates the zinc ion, the majority of the verified inhibitors contain a hydrogen bonding acceptor group (carboxylic, sulphonyl, amide) that interacts with Arg651, mimicking the substrate phosphate group.

### Exploring SAR of the salicylic acid-based PfThrRS inhibitors

**Fig 5:**
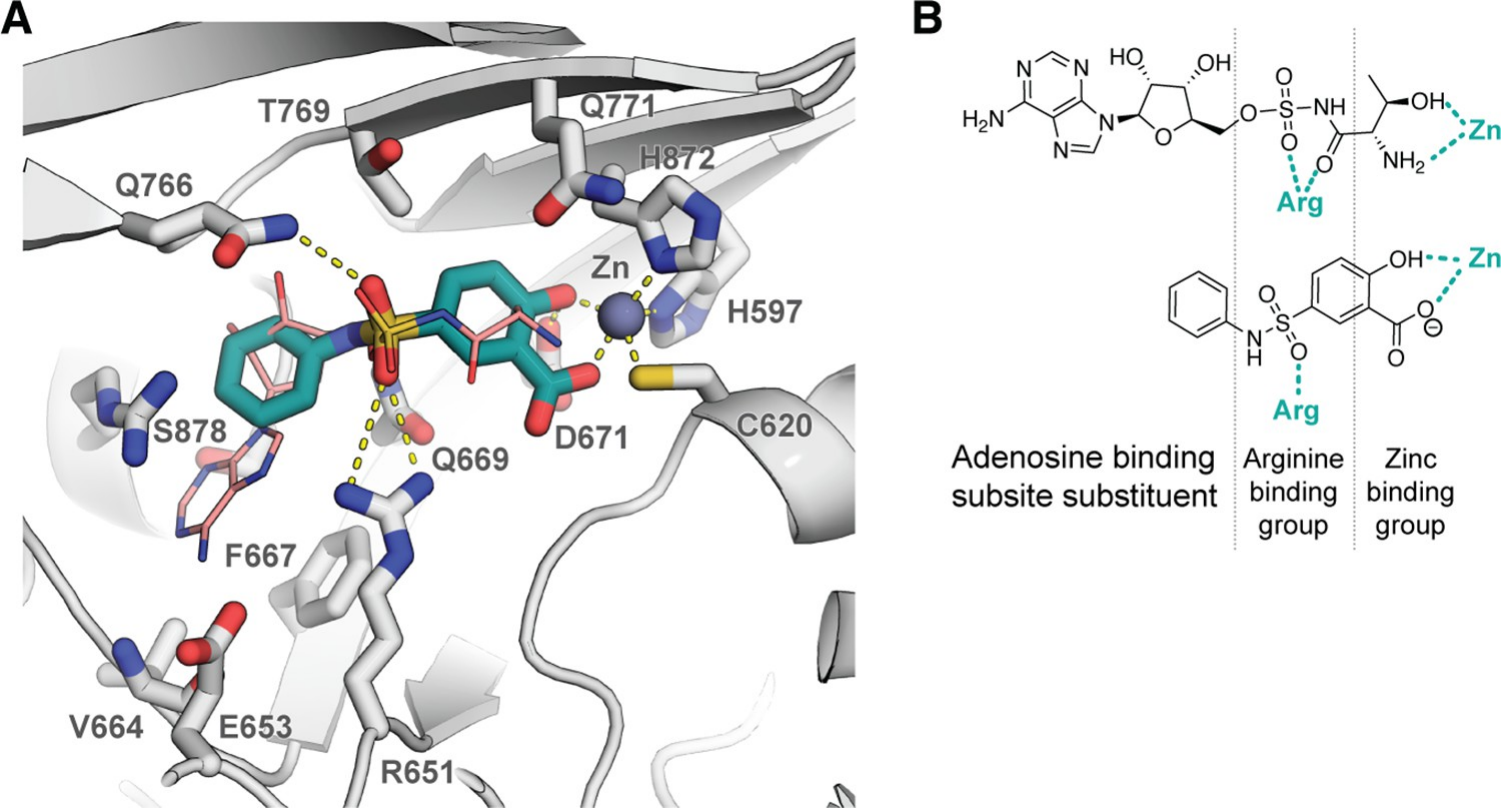
A docked pose of lead comp. 7 (teal) superimposed on ThrAMS (salmon; PDB ID: 1EVL). Binding site residue side chains are shown as grey sticks, hydrogen bonds are indicated as yellow dashed lines. Hydrogens are omitted for clarity. B Comparison of ThrAMS and comp. 7 functional groups forming key interactions in the binding site.

The salicylic acid-based compound MolPort-000-644-251 (**7**) was selected as a lead for optimisation due to its high ligand efficiency (0.35), structure simplicity, ease of synthetic modification, and commercial analogue availability. Compound optimisation was initiated by testing 83 commercially available analogues of **7** (see S1-S3 Tables in S1 Text) that contained modifications in three distinct regions: a) phenol and carboxylic acid–to explore various zinc binding groups; b) sulphonamide–to optimise interactions with Arg651; and c) aniline–to explore SAR of substituents that bind in the adenosine binding sub-site (Fig 5).

**Fig 6:**
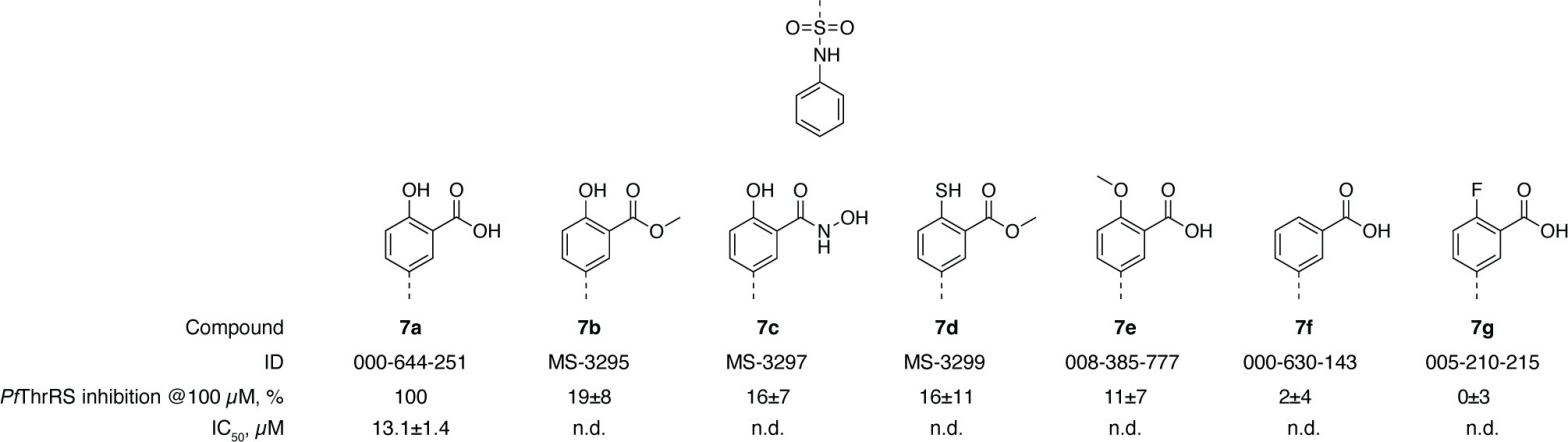
The SAR of the zinc coordinating group.

Any modifications to the zinc coordinating group were detrimental to compound activity, and none of the compounds tested showed activity comparable to the hit compound (see Fig 6). Some activity was maintained by the compounds that contained a hydrogen bond donor at the 2-position, enabling interactions with the zinc ion and Asp671. Nevertheless, these compounds inhibited *Pf*ThrRS activity by less than 20% at 100 μM concentration (IC_50_ values were determined only for compounds that inhibited *Pf*ThrRS by more than 50% at 100 μM concentration). Installation of additional substituents at benzoic acid deteriorated the activity even more, most likely due to steric clashes, as the threonine binding sub-site is narrow and it is not able to accommodate larger substituents.

**Fig 7:**
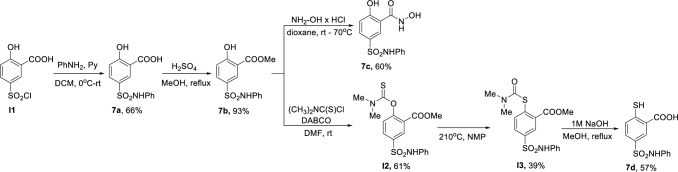
Scheme 1. Synthetic conditions for the synthesis of compounds 7a-d.

Compounds with various zinc coordinating groups (**7a-d**) were obtained according to Fig 7. Sulfonylation of aniline with commercially available sulfonyl chloride **I1** provided target compound **7a**. In the *Fischer* esterification reaction, ester **7b** was obtained, followed by an aminolysis reaction with hydroxylamine to deliver hydroxamic acid **7c**. *O*-Aryl thiocarbamate **I2** was synthesised in the reaction of phenol **7b** and dimethylthiocarbamoyl chloride. *S*-aryl thiocarbamate **I3** was obtained in *Newman–Kwart* rearrangement reaction. Finally, basic hydrolysis of compound **I3** yielded target compound **7d**.

**Fig 8:**
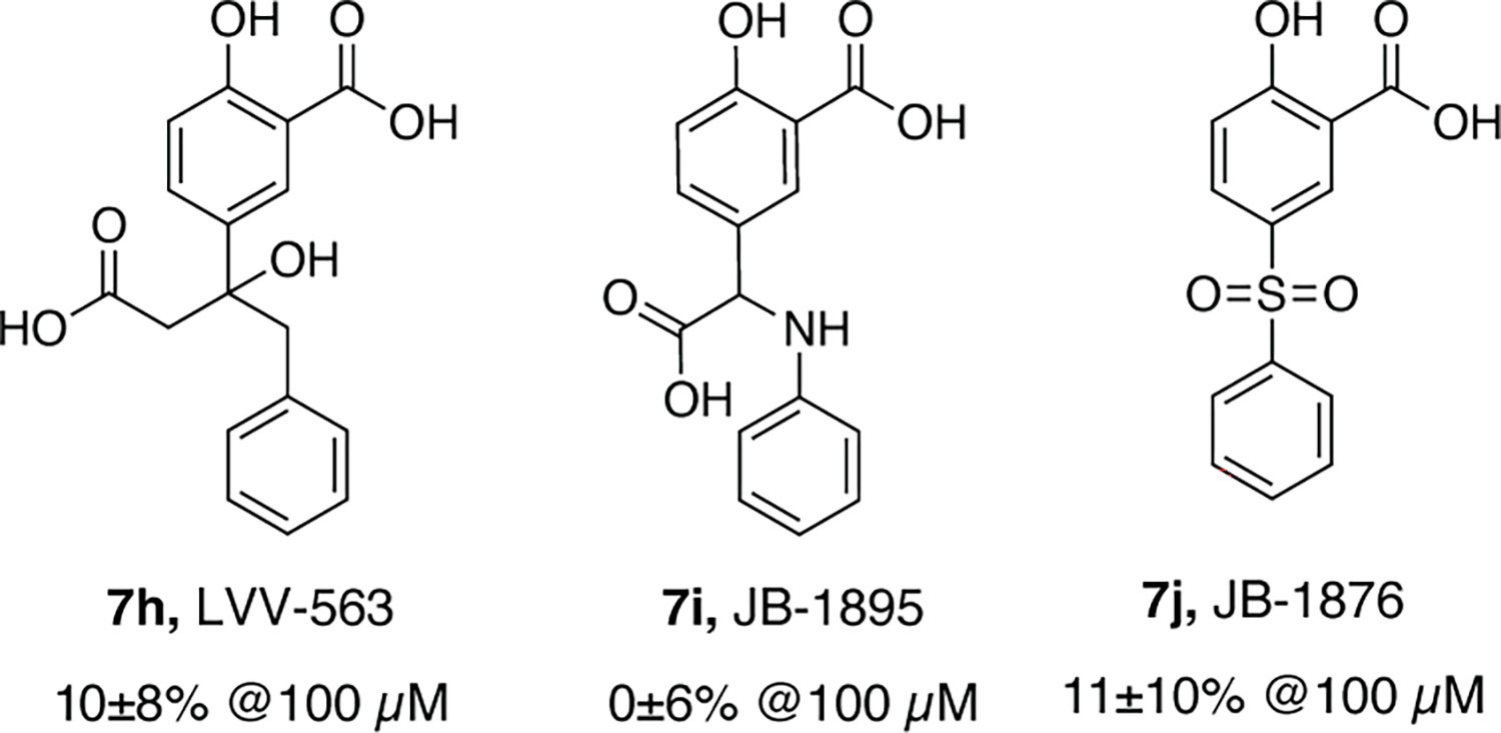
The SAR of arginine binding group.

Several compounds bearing modifications at the sulphonamide group (**7h-j**) were prepared to explore alternative arginine binding groups. These included compounds **7h,i**, which contained carboxylic acids capable of forming hydrogen bonds with Arg651, and analogue **7j** of parent hit **7a**, where the sulphonamide moiety was replaced with a sulphone group; however, no compound featuring an improved potency was identified (Fig 8). Most likely, this stems from a suboptimal alignment of the substituent with respect to Arg651, resulting in the absence of stabilising hydrogen bonding interactions with this key binding site residue.

**Fig 9:**
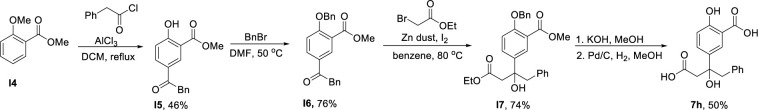
Scheme 2. Synthetic conditions for the synthesis of compound 7h.

Compound **7h** was prepared from ester **I4** as depicted in Fig 9. In the *Friedel-Crafts reaction* using AlCl_3_ ester **I4** was acylated, and then the hydroxyl group was protected to form derivative **I6**. After the *Reformatsky* reaction and subsequent hydrolyses of the methyl ester group and cleavage of the benzyl group final product **7h** was obtained.

**Fig 10:**
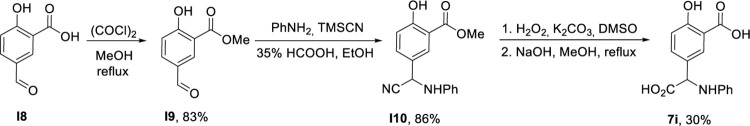
Scheme 3. Synthetic conditions for the synthesis of compound 7i.

Compound **7i** was synthesised from aldehyde **I8** in 4 steps as depicted in Fig 10. The first step involved an esterification reaction, followed by the *Strecker* reaction using aniline and TMSCN to form nitrile **I10**. Nitrile **I10** was converted to carboxylic acid **7i** in 2 steps: firstly, nitrile **I10** reacted with H_2_O_2_ and K_2_CO_3_ to form amide, followed by hydrolyses using NaOH.

**Fig 11:**
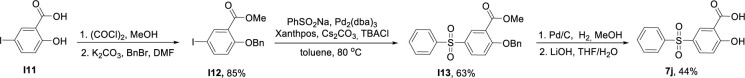
Synthetic conditions for the synthesis of compound 7j. Scheme 4.

Target compound **7j** was prepared from commercially available 2-hydroxy-5-iodobenzoic acid (**I11**) in 5 steps (Fig 11). Firstly, the carboxylic acid and hydroxyl group were protected, followed by Pd_2_(dba)_3_ catalysed coupling of aryl iodide **I12** with sodium benzenesulfinate. Finally, target compound **7j** was obtained after benzyl group hydrogenative cleavage and methyl ester group hydrolyses.

**Fig 12:**
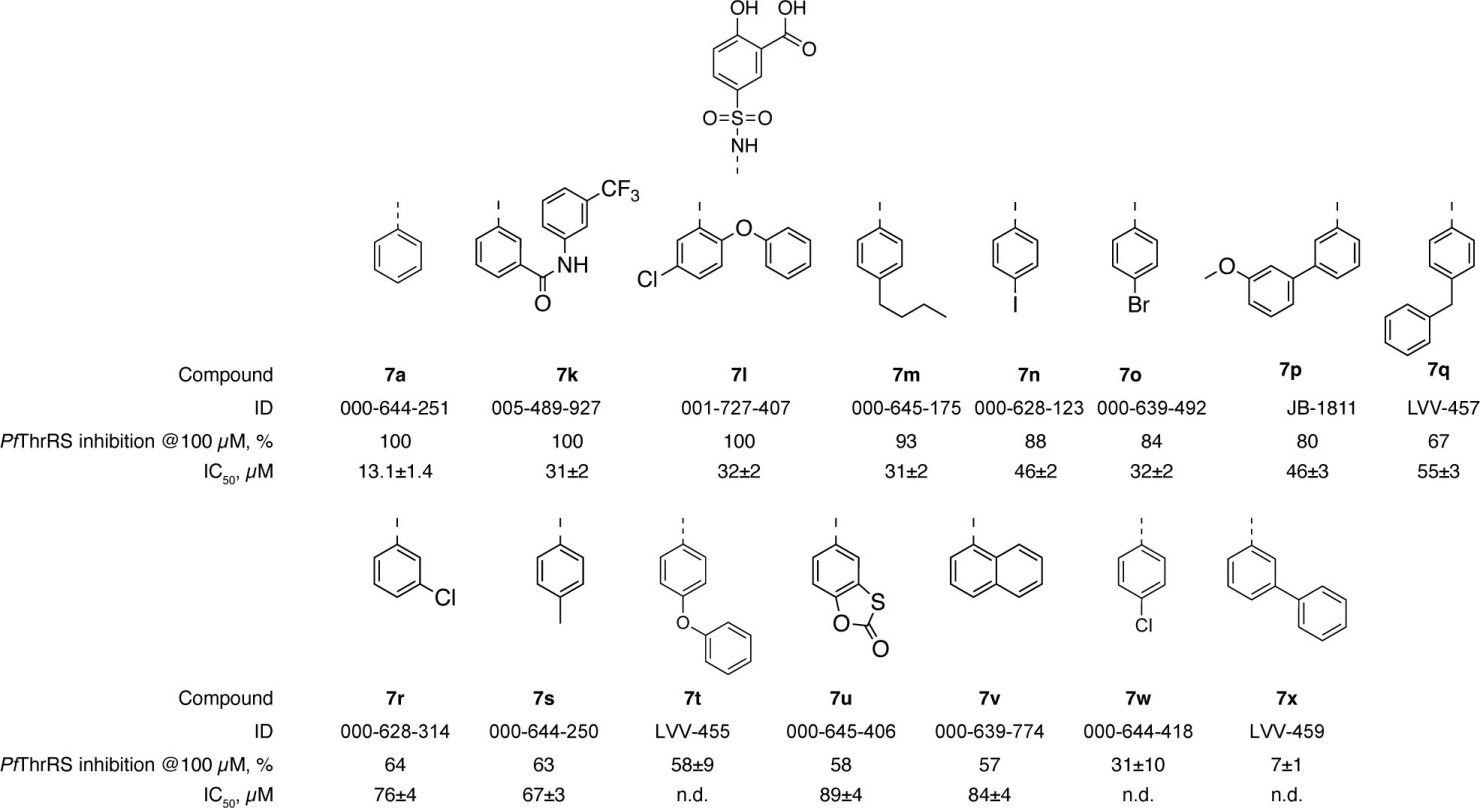
The SAR of adenosine binding sub-pocket substituent.

**Fig 13:**
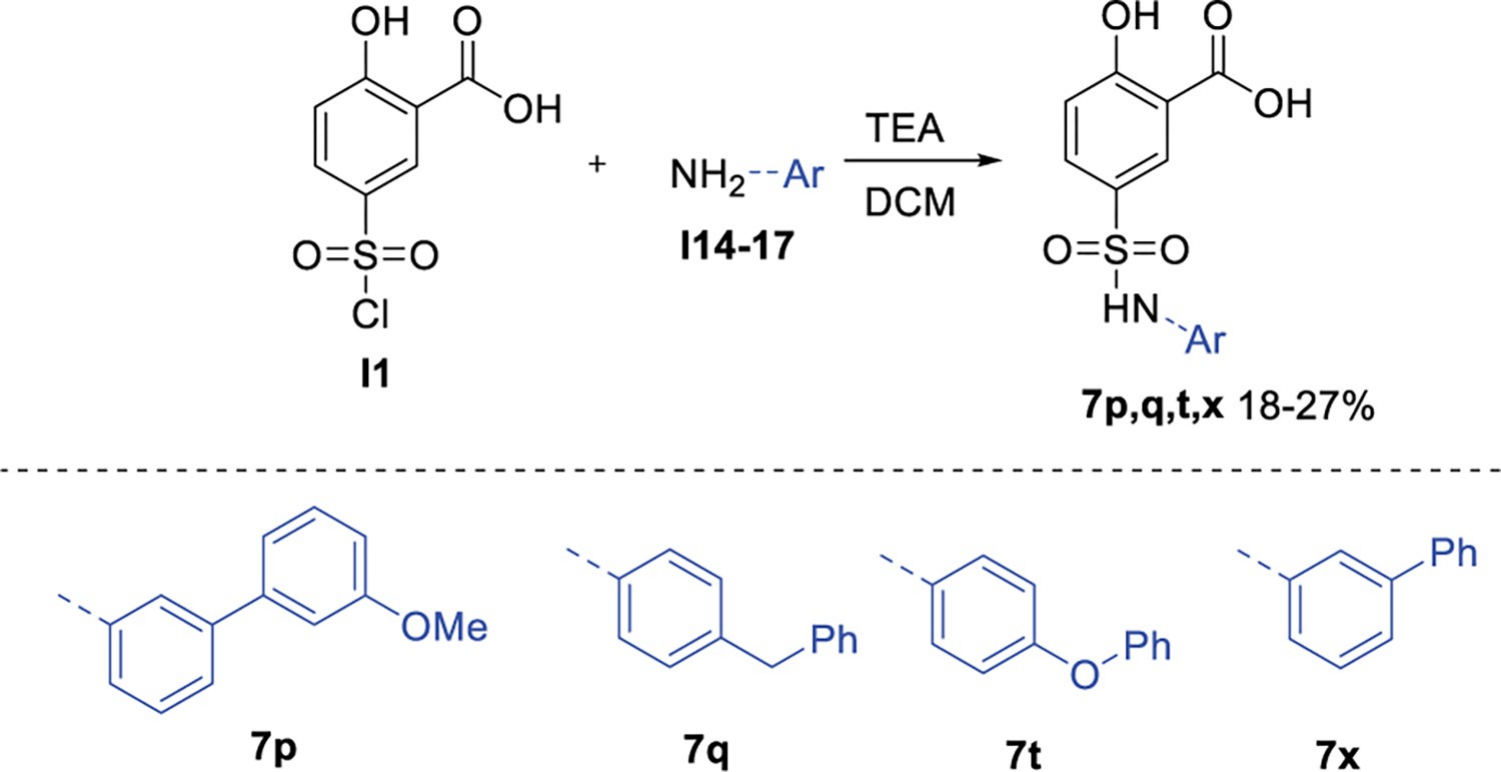
Scheme 5. General procedure for sulfonylation of anilines 21a-e.

Once we established that modifications at the zinc ion and Arg651 binding groups are not tolerated, lead compound optimisation focused on filling the adenosine-binding sub-pocket. Based on the available crystal structure of the ThrAMS-*Ec*ThrRS complex, various substituents were installed, mostly at the 3- and 4-position of the phenylsulphamoyl group. A total of 120 compounds with modifications at the phenylsulphamoyl group were either purchased from commercial vendors or synthesized (see the full list of compounds in S1 Table in S1 Text). The majority of the commercially available **7a** analogues contained minor substituents (1–2 non-hydrogen atoms) at the phenylsulphamoyl group, whereas the docking model indicated that compounds containing additional aromatic ring could form stacking interactions with Phe667, and yield more potent inhibitors. Taking this into account, compounds containing aromatic substituents in positions 3 and 4 of the phenylsulphamoyl group were prepared, and sub 100 μM *Pf*ThrRS inhibitors were identified (see Fig 12). The synthesis of compounds **7p, 7q, 7t** and **7x** was accomplished as depicted in general Fig 13. Sulfonylation of corresponding anilines **I14-17** with commercially available sulfonyl chloride I**1** using triethylamine (TEA) base gave sulfamoyl salicylic acid analogues **7p,q,t,x**. Reaction conditions and yields were not optimised. To be noted, anilines **I15,16** are commercially available, while anilines **I14,17** were prepared according to the literature [24, 25].

**Fig 14:**
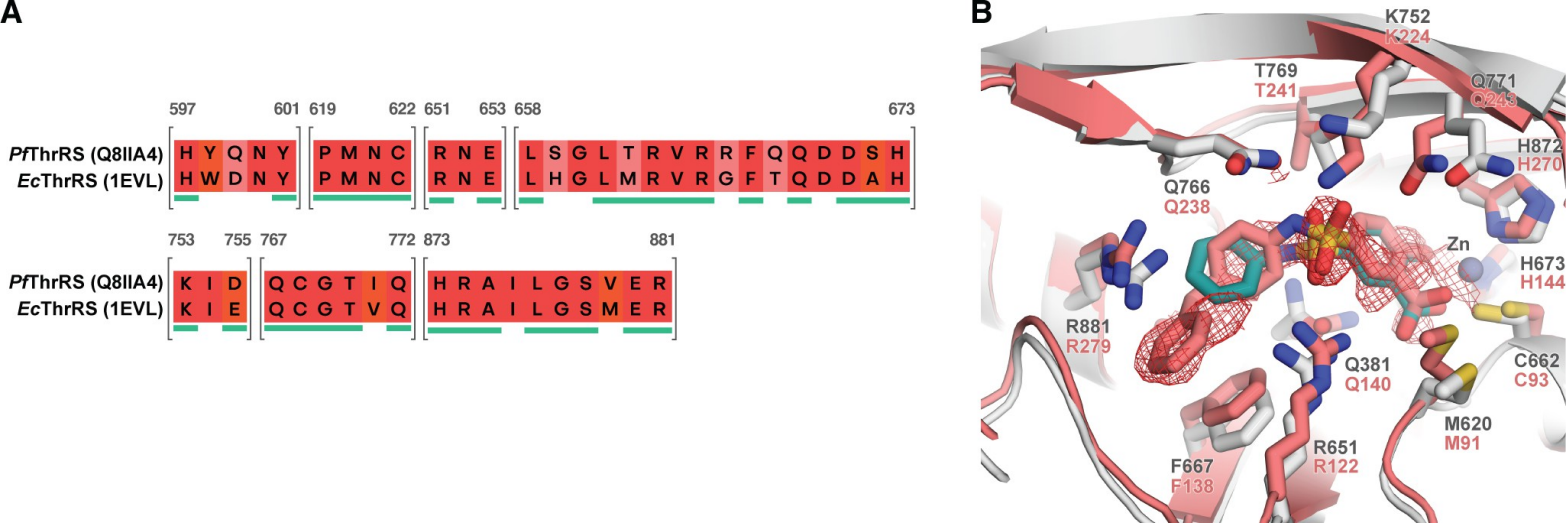
A sequence alignment of PfThrRS and EcThrRS binding sites. Shading indicates the conservation of residues with fully conserved positions shaded in dark red. Residue numbers on top refer to the PfThrRS. Residues within 5 Å from the ligand (ThrAMS) are underlined in teal. B Compound 7q (salmon) modelled in the EcThrRS binding site electron density (mF_O_-DF_C_, contoured at 1.5 σ). Overlayed is docked pose of screening hit 7a (teal). Binding site residue side chains are shown as grey (PfThrRS) and salmon (EcThrRS) sticks; hydrogens are omitted for clarity.

Attempts were made to co-crystallise these inhibitors with *Pf*ThrRS to enable experimental structure-based drug design; however, no crystals suitable for X-ray analysis were obtained for the *Plasmodium falciparum* ThrRS isoform. Since crystallisation condition protocols for the *E*. *coli* ThrRS were available [10, 16, 22, 26–28], and since the ThrRSs of the two organisms share 94.7% sequence identity in the binding site (within 3 Å from ThrAMS; 89.2% within 5 Å; see binding site sequence alignment in Fig 14A), attempts were made to co-crystallise the identified inhibitors with the *Ec*ThrRS, and use this information to guide compound optimisation. Crystallisation experiments resulted in the determination of the crystal structure of the *Ec*ThrRS-**7q** complex, which was resolved at 2.08 Å resolution. Structure analysis confirmed the docking predictions that the compound binds in a threonine-binding subsite and that the salicylic acid hydroxyl and carboxylic groups coordinate the zinc ion (the heavy atom RMSD value between the crystallised and docked pose is 2.54 Å; see Fig 14B). The binding mode of the zinc-coordinating phenol/carboxylic acid group was identical when comparing the experimental crystal structure and the docked model. However, there were minor differences in the conformation of the sulphonamide group (see Fig 14B). The sulphonamide group was slightly twisted with respect to the docked pose, and besides binding to Arg651, it simultaneously forms interactions also with Lys752, Gln766 and Gln 771. The discrepancy between the binding mode of the resolved and the docked sulphonamide group was the result of Lys752, Gln766 and Gln 771 side chain mobility, as the conformations showed by these residues were different from those in the complex with ThrAMS.

## Conclusions

Despite the availability of the structural information on the ligand binding mode, targeted incorporation of hydrogen bond donors and acceptors to mimic the adenine of the substrate did not yield compounds with substantially improved potency. The highest *Pf*ThrRS inhibition potency within this series was achieved by compounds **7k,l,p,q,t**, which introduced stacking interactions with Phe667, similar to those of ThrAMS, or ionic interactions with Arg881 and/or Ser878 (comp. **7n,o,r**). Nevertheless, the initial virtual screening hit **7** remained one of the most active compounds within this series. Moreover, the high *Pf*ThrRS and *Hs*ThrRS sequence identity in the binding site (88% within 5 A from the inhibitor) suggests that it would be a challenging task to tune these salicylic acid-based compounds as selective *Pf*ThrRS inhibitors.

## Materials and methods

### Homology modelling

The homology models of *Plasmodium falciparum* ThrRS (Uniprot ID: Q8IIA4, 556–895) were based on *Escherichia coli* ThrRS crystal structures 1EVL [22] and 4P3N [10], and were generated using Schrodinger Maestro software package [29]. Pairwise sequence alignment was performed using Muscle [30] and the model was built using a knowledge-based homology modelling tool. The penalty for the gap opening was 10.0 and 0.2 for the gap extension. The non-template loops of the obtained homology models were refined using Prime [31]. VSGB solvation model [32] and OPLS4 [33] force field were used to refine loops that were longer than 3 residues. The sequence alignment files and homology model coordinates are attached as supporting data (S1 Data).

### High-Throughput Virtual Screening (HTVS)

MolPort in-stock drug-like compound library of ~6.3M comp. (2021) and collection of open natural products COCONUT [23] (~400k comp., 2021) were prepared using LigPrep [34] by desalting the molecules, generating possible tautomers and ionisation states at pH 7.0± 2.0. The stereochemistry of the compounds was retained as specified in the library.

The prepared libraries were docked in the homology models created. Homology models were prepared for docking using Maestro Protein Preparation Wizard by optimising loops using Prime, adjusting side chain protonation states at pH 7.0, and minimising heavy atoms with convergence up to 0.30 Å. Molecular docking was performed using Glide [35], with scaling of the van der Waals radii set to 0.9 for protein and ligand heavy atoms, and docking compounds flexibly. The top-scoring 3000 compounds were clustered into 300 representative compounds by calculating the Linear Fingerprints from Daylight invariant atom types and evaluating compound similarity using Tanimoto similarity metrics. The top-scoring compound was retained for each cluster. The top-ranked 300 representative compounds for each homology model were visually inspected for their ability to form hydrogen bonds similar to those established by the co-crystallised ligand, with molecules showing internal strains or unsatisfied hydrogen bond donors being deprioritised. A total of 40 drug-like and 18 natural product-like compounds were selected and purchased from MolPort. Docked poses were visualised using PyMOL [36].

### Protein expression and purification

Protein production was conducted as described elsewhere [21]. *Escherichia coli* ThrRS was over-expressed in *E*. *coli* BL21 (DE3) cells. The cells were grown in a Luria–Bertani (LB) medium at 37°C until OD_600_ = 0.8, when expression was induced with 0.5 mM IPTG for 16 h at 20°C. *Plasmodium falciparum* ThrRS was expressed in BL21(DE3) cells at 16°C for 20 h using 0.5 mM IPTG. In both cases, cells were then harvested by centrifugation and resuspended in lysis buffer (20 mM Tris, pH 8.0, 300 mM NaCl, 1 mM TCEP and EDTA free protease inhibitors). After sonication and ultracentrifugation, proteins were found predominantly in the soluble fraction. *Ec*ThrRS protein was first purified by immobilised metal affinity chromatography (IMAC) with a 5 mL His-Trap column (GE Healthcare) and eluted with a linear imidazole gradient (0–0.5 M) in 15 column volumes (CV). The eluted fractions were then buffer-exchanged in 20 mM Tris, pH 8.0, and 1 mM TCEP and then loaded in a 5 mL ion exchange Q-HP column (GE Healthcare) and eluted with a linear NaCl gradient (0–1M) in 20 column volumes (CV). Selected fractions were then loaded in a Superdex 200 26/60 (Cytiva) in 20 mM Tris, pH 8.0, 300 mM NaCl, and 1 mM TCEP and purified by size exclusion chromatography. No cleavage of the His-tag was done, and the yield was 7.5 mg/L (*Ec*ThrRS) and 19 mg/L (*Pf*ThrRS).

### *In vitro* PfThrRS inhibition assay

The bioassay protocol was adapted from the literature [19, 21]. The aminoacylation activity of *Pf*ThrRS in the presence of compounds was determined by measuring ATP consumption in the enzymatic reaction by using recombinant *Pf*ThrRS and *P*. *falciparum* cell extract as a source of t-RNA. ATP consumption was determined by Kinase-Glo® Reagent (Promega Inc.). The luminescence was read on a Tecan Infinite M1000 microplate reader. The inhibitory rate was measured in three independent assays. Borrelidin was obtained from commercial sources and used as a reference compound.

### Protein crystallography

*Ec*ThrRS crystallisation screens were done using the hanging drop method, as described elsewhere [21]. In brief, to crystallise the *Ec*ThrRS–**7q** complex, the protein solution (10–15 mg/mL) was pre-mixed with 2 mM **7q** and incubated for 3 h at 4°C. The complex was then crystallised by mixing 1 μL of protein solution with 1 μL of 10% (w/v) PEG 4000, 18% (v/v) MPD, and 0.1 M sodium citrate pH 5.9 crystallisation buffer. Prior to X-ray diffraction data collection, crystals were flash-frozen in liquid nitrogen using 25% glycerol as a cryoprotectant. Diffraction data were collected at the PX3 beamline of the Swiss Light Source (SLS). Data were integrated and scaled within the DIALS [37] suite using XIA2 [38]. The structures were determined by molecular replacement using the *Ec*ThrRS structure (PDB: 8OU8) as the starting model, and the program PHASER [39]. After corrections for bulk solvent and overall B values, data were refined by iterative cycles of positional refinement and TLS refinement with REFMAC [40] and model building with COOT [41] from the CCP4 suite [42]. Final coordinates have been deposited in the Protein Data Bank (PDB, accession code: 8QIC). Data collection and model statistics are shown in S4 Table in S1 Text.

### Organic synthesis

#### General experimental procedures

^1^H, ^13^C NMR spectra were recorded on 300 MHz and 400 MHz Bruker spectrometers using the residual solvent peak (7.26 ppm and 77.16 ppm for ^1^H and ^13^C in CDCl_3_; 3.31 ppm and 49.00 ppm for 1H and ^13^C in MeOD; 2.50 ppm and 39.52 ppm for ^1^H and ^13^C in DMSO-d6) as an internal reference. Column chromatography was performed using Kieselgel silica gel (35–70 μm and 60–200 μm). Reverse-phase chromatography was performed using KP-C18-HS SNAP Biotage cartridges on a Biotage Isolera One purification system. Thin layer chromatography (TLC) was performed on silica gel using Merck TLC Silica gel 60 F254 aluminium sheets and was visualized by UV lamp, staining with KMnO_4_. HRMS were obtained on a Waters Synapt G2-Si hybrid quadrupole time-of-flight (TOF) mass spectrometer equipped with an electron spray ion source (ESI). Analytical HPLC data was obtained using Waters Alliance LC systems equipped with a 2695 separation module with Adamas C18 4.6 x 150 mm or Apollo 5 μm C18 4.6 x 150 mm column and Waters 2489 dual absorbance detector. Reagents and starting materials were purchased from commercial sources and used as received. Throughout the experiments, no unexpected or unusually high safety hazards were encountered.

#### 2-Hydroxy-5-(N-phenylsulfamoyl)benzoic acid (7a)

Is known in the literature [43]. ^1^H NMR (400 MHz, DMSO-*d*_6_) δ 10.18 (s, 1H), 8.15 (d, *J* = 2.4 Hz, 1H), 7.78 (dd, *J* = 8.8, 2.5 Hz, 1H), 7.28–7.20 (m, 2H), 7.10–6.99 (m, 4H). ^13^C NMR (101 MHz, DMSO) δ 170.44, 163.98, 137.64, 133.35, 129.86, 129.29, 124.37, 120.43, 118.29, 113.74. HRMS (ESI) for C_13_H_12_NO_5_S [M+H]+, calcd 294.0436, found 294.0440. Purity by HPLC analysis on Apollo C18: at 210 nm—99.83%; at 254 nm—99.85%.

#### Methyl 2-hydroxy-5-(N-phenylsulfamoyl)benzoate (7b)

Is known in literature [44]. ^1^H NMR (400 MHz, MeOD) δ 8.22 (d, *J* = 2.4 Hz, 1H), 7.80 (dd, *J* = 8.9, 2.4 Hz, 1H), 7.25–7.17 (m, 2H), 7.10–7.03 (m, 3H), 7.01 (d, *J* = 8.9 Hz, 1H), 3.95 (s, 3H). ^13^C NMR (101 MHz, Methanol-*d*_4_) δ 170.49, 165.61, 138.82, 134.99, 131.76, 131.20, 130.20, 125.98, 122.57, 119.37, 113.63, 53.38. HRMS (ESI) for C_14_H_14_NO_5_S [M+H]+, calcd 308.0593, found 308.0598. Purity by HPLC analysis on Apollo C18: at 210 nm—99.95%; at 254 nm—100%.

#### N,2-dihydroxy-5-(N-phenylsulfamoyl)benzamide (7c)

Dissolve hydroxylamine hydrochloride (45 mg, 0.65 mmol) and NaOH (39.04 mg, 0.98 mmol) in water (3 mL). Add a solution of methyl 2-hydroxy-5-(*N*-phenylsulfamoyl)benzoate (**7b**) (100 mg, 0.33 mmol) in 1,4-dioxane (2 mL) to the solution dropwise. Stir the mixture at rt for 12 hours, then 5 hours at 70°C. Acidify the solution with a 1 M water solution of hydrochloric acid to pH 5 to precipitate colorless crystalline. Filter the formed filtered precipitate. Wash the mixture with cold (5°C) water (5 ml) and dry in vacuo for 3 h to obtain product (60 mg, 60% yield). ^1^H NMR (400 MHz, DMSO-*d*_6_) δ 12.44 (bs, 1H), 11.32 (bs, 1H), 10.16 (s, 1H), 9.37 (bs, 1H), 8.14 (d, *J* = 2.5 Hz, 1H), 7.68 (dd, *J* = 8.7, 2.5 Hz, 1H), 7.28–7.18 (m, 2H), 7.12–7.04 (m, 2H), 7.04–6.95 (m, 2H). ^13^C NMR (101 MHz, DMSO-*d*_6_) δ 163.50, 161.18, 137.76, 131.11, 129.66, 129.14, 128.13, 123.94, 119.89, 117.76, 116.29. HRMS (ESI) for C_13_H_13_N_2_O_5_S [M+H]+, calcd 309.0545, found 309.0554. Purity by HPLC analysis on Apollo C18: at 210 nm—94.02%; at 254 nm—96.03%.

#### Methyl 2-((dimethylcarbamothioyl)oxy)-5-(N-phenylsulfamoyl)benzoate (I2)

Add 1,4-diazabicyclo[2.2.2]octane (82 mg, 0.73 mmol) to methyl 2-hydroxy-5-(*N*-phenylsulfamoyl)benzoate (**7b**) (149 mg, 0.49 mmol) in dry DMF (2 ml) under argon. Stir the mixture at room temperature for 5 minutes. Add *N*,*N-*dimethylthiocarbamoyl chloride (90.37 mg, 0.731 mmol) to the mixture in one portion. Stir the reaction mixture overnight. Evaporate reaction mixture to dryness, then dilute with EtOAc (10 mL), wash with 1M NaOH aqueous solution (2 x 10 mL), then sat. NaCl aqueous solution (1 x 10 mL). Dry organic layer over Na_2_SO_4_, and concentrate under reduced pressure. Purify the reaction mixture by chromatography (silica gel, 50/50 v/v petrolether/EtOAc) to give product (117 mg, 61% yield). ^1^H NMR (400 MHz, MeOD) δ 8.28 (d, *J* = 2.4 Hz, 1H), 7.88 (dd, *J* = 8.5, 2.4 Hz, 1H), 7.28–7.18 (m, 3H), 7.14–7.05 (m, 3H), 3.82 (s, 3H), 3.40 (s, 3H), 3.37 (s, 3H). LC-MS: m/z calculated for C_17_H_19_N_2_O_5_S_2_ [M+ H^+^]: 395; found 395.

#### Methyl 2-((dimethylcarbamoyl)thio)-5-(N-phenylsulfamoyl)benzoate (I3)

The solution of methyl 2-((dimethylcarbamothioyl)oxy)-5-(*N*-phenylsulfamoyl)benzoate (**I2**) (117 mg, 0.30 mmol) in NMP (2 mL) was heated for 2 hours under argon atmosphere at 210°C. After completion of the reaction as monitored by TLC, reaction mixture was cooled to rt and EtOAc (10 mL) was added, followed by sat. NaCl aqueous solution (20 mL). Organic layer was washed with water, dried over Na_2_SO_4_, and concentrated under reduced pressure. The reaction mixture was purified by chromatography (silica gel, 50/50 v/v petrolether/EtOAc) to give product (45 mg, 39% yield). ^1^H NMR (400 MHz, CDCl_3_) δ 8.27 (dd, *J* = 2.1, 0.5 Hz, 1H), 7.75 (dd, *J* = 8.3, 2.1 Hz, 1H), 7.70 (dd, *J* = 8.2, 0.5 Hz, 1H), 7.28–7.21 (m, 2H), 7.17–7.10 (m, 1H), 7.10–7.03 (m, 2H), 3.88 (s, 3H), 3.13 (bs, 3H), 3.03 (bs, 3H). LC-MS: m/z calculated for C_17_H_19_N_2_O_5_S_2_ [M+ H^+^]: 395; found 395.

#### 2-Mercapto-5-(N-phenylsulfamoyl)benzoic acid (7d)

A solution of methyl 2-((dimethylcarbamoyl)thio)-5-(*N*-phenylsulfamoyl)benzoate (**I3**) (45 mg, 0.11 mmol) in degassed 1.0 M NaOH/MeOH/H_2_O (1:1:1, 3 mL) was refluxed under argon atmosphere for 1 h. The reaction mixture was cooled in ice, and conc. HCl (0.5 mL) was added. A white precipitate was formed, filtered, and washed extensively with water to give product (20 mg, 57% yield). Unstable under characterization conditions, forms dimer. HRMS (ESI) for C_13_H_12_NO_4_S_2_ [M+H]+, calcd 310.0208, found 310.0213.

#### Methyl 2-hydroxy-5-(2-phenylacetyl)benzoate (I5)

To the solution of methyl 2-methoxybenzoate (**I4**) (1 ml, 6.98 mmol) in DCM (20 mL) under argon were successively added phenylacetyl chloride (1.85 ml, 14.00 mmol) and anhydrous AlCl_3_ (3.72 g, 27.92 mmol) at 0°C. The reaction mixture was refluxed for 5 hours, then cooled to 0°C and a solution of 1 M HCl was added dropwise until the solution became clear. The reaction mixture was extracted with DCM, dried over Na_2_SO_4_, and concentrated under reduced pressure. The reaction mixture was purified by chromatography (silica gel, 75/25 v/v n-hexane/ EtOAc) to give product (865 mg, 46% yield). ^1^H NMR (300 MHz, CDCl_3_) δ 11.21 (s, 1H), 8.55 (d, *J* = 2.3 Hz, 1H), 8.11 (dd, *J* = 8.8, 2.3 Hz, 1H), 7.34–7.23 (m, 5H), 7.01 (d, *J* = 8.8 Hz, 1H), 4.23 (s, 2H), 3.98 (s, 3H). LC-MS: m/z calculated for C_16_H_14_O_4_ [M+ H^+^]: 271; found 271.

#### Methyl 2-(benzyloxy)-5-(2-phenylacetyl)benzoate (I6)

To the reaction mixture of methyl 2-hydroxy-5-(2-phenylacethyl)benzoate (**I5**) (865 mg, 3.20 mmol) and K_2_CO_3_ (885 mg, 6.40 mmol) solution in DMF (7 mL) was added dropwise BnBr (0.38 mL, 3.20 mmol) at rt. The reaction mixture was heated at 50°C for overnight. After cooling, the reaction mixture was diluted with EtOAc, washed with water, then washed with brine, dried over Na_2_SO_4_, and concentrated under reduced pressure. The reaction mixture was purified by chromatography (silica gel, 75/25 v/v n-hexane/EtOAc) to give product (0.819 g, 71% yield). ^1^H NMR (300 MHz, CDCl_3_) δ 8.52 (d, *J* = 2.4 Hz, 1H), 8.10 (dd, *J* = 8.8, 2.4 Hz, 1H), 7.49–7.25 (m, 10H), 7.05 (d, *J* = 8.8 Hz, 1H), 5.26 (s, 2H), 4.25 (s, 2H), 3.93 (s, 3H). LC-MS: m/z calculated for C_23_H_20_O_4_ [M+ H^+^]: 361; found 361.

#### Methyl 2-(benzyloxy)-5-(4-ethoxy-2-hydroxy-4-oxo-1-phenylbutan-2-yl)benzoate (I7)

The mixture of methyl 2-benzyloxy-5-phenylacethylbenzoate (**I6**) (250 mg, 0.69 mmol), Zn dust (91 mg, 1.39 mmol) and catalytic amount of I_2_ in benzene (1.8 mL) was refluxed for 1h. Then a solution of ethyl bromoacetate (0.12 mL, 1.04 mmol) in dry benzene (1.7 mL) was added dropwise to the mild refluxing reaction mixture under argon. The reaction mixture was reflux for 6-7h. After completion of the reaction (monitored by UPLC), reaction mixture was cooled at 0°C, quenched with 10% HCl, extracted with EtOAc, washed with water, washed with brine, dried over Na_2_SO_4_ and concentrated under reduced pressure. The reaction mixture was purified by chromatography (silica gel, 75/25 v/v n-hexane/EtOAc) to give product (230 mg, 74% yield). ^1^H NMR (300 MHz, CDCl_3_) δ 7.82 (d, *J* = 2.5 Hz, 1H), 7.53–7.45 (m, 2H), 7.43–7.29 (m, 4H), 7.24–7.16 (m, 3H), 7.04–6.97 (m, 2H), 6.93 (d, *J* = 8.8 Hz, 1H), 5.17 (s, 2H), 4.46 (s, 1H), 4.00 (q, *J* = 7.1 Hz, 2H), 3.90 (s, 3H), 3.10–2.92 (m, 3H), 2.75 (d, *J* = 16.0 Hz, 1H), 1.09 (t, *J* = 7.2 Hz, 3H).

#### 5-(1-Carboxy-2-hydroxy-3-phenylpropan-2-yl)-2-hydroxybenzoic acid (7h)

Methyl 2-(benzyloxy)-5-(4-ethoxy-2-hydroxy-4-oxo-1-phenylbutan-2-yl)benzoate (**I7**) (300 mg, 0.67 mmol) was dissolved in MeOH (10 mL), added KOH (300 mg, 5.44 mol) and stirred at 40°C for 6–8 h (monitored by UPLC). After completion of the reaction, the mixture was concentrated on rotavapor, diluted with water and acidified to pH = 4 with aq. HCl, extracted with DCM (3 x 20 mL). The combined DCM layer was dried over Na_2_SO_4_, filtered and the solvent was evaporated. Crude product without purification was dissolved in MeOH (7 ml). To the solution was added 10% Pd/C (24 mg) and the reaction mixture was hydrogenated (2 bar pressure of H_2_) for 2 h at rt. After completion of the reaction (UPLC control), the reaction mixture was filtrated through Celite and evaporated. The residue was purified by chromatography (C18 silica gel, 95/5–0/100 v/v H_2_O/MeCN) to give the product (106 mg, 50% yield). ^1^H NMR (300 MHz, MeOD) δ 7.82 (d, *J* = 2.4 Hz, 1H), 7.44 (dd, *J* = 8.7, 2.5 Hz, 1H), 7.15 (dd, *J* = 4.9, 1.9 Hz, 3H), 6.99 (dd, *J* = 6.6, 2.9 Hz, 2H), 6.83 (d, *J* = 8.7 Hz, 1H), 3.09 (s, 2H), 2.95 (d, *J* = 15.5 Hz, 1H), 2.76 (d, *J* = 15.5 Hz, 1H).^13^C NMR (101 MHz, MeOD) δ 175.48, 173.57, 162.00, 137.87, 137.59, 134.27, 132.00, 128.72, 128.65, 127.41, 117.50, 112.96, 75.95, 49.96, 44.83. Not stable under HRMS conditions. LC-MS: m/z calculated for C_17_H_16_O_6_ [M-H^+^]: 315; found 315. Purity by HPLC analysis on Adamas C18: at 210 nm—99.39%; at 254 nm—95.53%.

#### Methyl 5-formyl-2-hydroxybenzoate (I9)

To the mixture of 5-formylsalicylic acid (**I8)** (1.00 g, 6.02 mmol) in MeOH (24 mL) was added dropwise oxalyl chloride (1.58 mL, 18.06 mmol) at 0°C. The reaction mixture was heated to reflux and stirred for 5 hours. The reaction mixture was concentrated and dried in vacuo to give the product (960 mg, 89%). The compound is previously described in the literature [45]. ^1^H NMR (300 MHz, CDCl_3_) δ 11.35 (s, 1H), 9.88 (s, 1H), 8.39 (d, *J* = 2.1 Hz, 1H), 8.01 (dd, *J* = 8.7, 2.2 Hz, 1H), 7.11 (d, *J* = 8.7 Hz, 1H), 4.01 (s, 3H).

#### Methyl 5-(cyano(phenylamino)methyl)-2-hydroxybenzoate (I10)

The mixture of methyl 5-formyl-2-hydroxybenzoate (**I9**) (815 mg, 4.52 mmol), aniline (0.41 mL, 4.52 mmol), trimethylsilyl cyanide (0.62 mL, 4.98 mmol), and 37% aqueous formic acid (0.13 mL, 1.13 mmol) was stirred vigorously in EtOH (18 ml) at rt. After completion of the reaction as monitored by TLC, reaction mixture was evaporated and diluted with EtOAc, washed with water, dried over Na_2_SO_4_, and concentrated under reduced pressure. The reaction mixture was purified by chromatography (silica gel, 70/30 v/v petrolether/EtOAc) to give product (1.10 g, 86% yield). ^1^H NMR (300 MHz, DMSO) δ 10.61 (s, 1H), 8.01 (d, *J* = 2.6 Hz, 1H), 7.69 (dd, *J* = 8.7, 2.6 Hz, 1H), 7.22–7.12 (m, 2H), 7.09 (d, *J* = 8.6 Hz, 1H), 6.81 (d, *J* = 7.6 Hz, 2H), 6.73 (t, *J* = 7.3 Hz, 1H), 6.65 (d, *J* = 9.5 Hz, 1H), 5.96 (d, *J* = 9.5 Hz, 1H), 3.91 (s, 3H). ^13^C NMR (101 MHz, DMSO-*d*_6_) δ 168.50, 160.02, 145.86, 134.45, 129.05, 129.00, 126.03, 119.49, 118.48, 118.19, 113.89, 113.46, 52.60, 47.46. HRMS (ESI) for C_16_H_15_N_2_O_3_ [M+H]+, calcd 283.1083, found 283.1084.

#### 5-(Carboxy(phenylamino)methyl)-2-hydroxybenzoic acid (7i)

To the mixture of methyl 5-(cyano(phenylamino)methyl)-2-hydroxybenzoate (**I10**) (200 mg, 0.71 mmol) and K_2_CO_3_ (98 mg, 0.71 mmol) in DMSO (0.9 mL) was added 30% H_2_O_2_ (0.11 ml, 1.06 mmol) at 0°C. The mixture was warmed to rt and stirred for 2 hours. The precipitate was collected by filtration, washed with cold water and dried *in vacuo*. The residue was dissolved in a mixture of MeOH/H_2_O (4:1, 5 mL) and NaOH (113 mg, 2.83 mmol) was then added. This reaction mixture was refluxed for 5 hour and concentrated. Water (30 mL) was added and the resulting mixture was extracted with EtOAc (20 mL). The aqueous phase was acidified to pH = 4 with conc. HCl, and extracted with DCM (3 x 20 mL). The combined DCM layer was washed with brine, dried over Na_2_SO_4_, filtered and the solvent was evaporated to give product (60 mg, 30% yield). ^1^H NMR (300 MHz, DMSO-*d*_6_) δ 11.56–10.89 (brs, 1H), 7.92 (d, *J* = 2.4 Hz, 1H), 7.63 (dd, *J* = 8.6, 2.4 Hz, 1H), 7.07–6.99 (m, 2H), 6.95 (d, *J* = 8.6 Hz, 1H), 6.64 (d, *J* = 7.6 Hz, 2H), 6.54 (t, *J* = 7.3 Hz, 1H), 5.06 (s, 1H). ^13^C NMR (101 MHz, MeOD) δ 175.07, 173.31, 162.99, 147.83, 135.77, 130.60, 130.33, 130.01, 119.00, 118.50, 114.87, 113.98, 61.25. HRMS (ESI) for C_14_H_12_NO_3_ [M—CO_2_H]+, calcd 242.0817, found 242.0829. LC-MS: m/z calculated for C_15_H_14_NO_5_ [M+ H^+^]: 288; found 288. Purity by HPLC analysis on Adamas C18: at 210 nm—93.26%; at 254 nm—92.95%.

#### Methyl 2-(benzyloxy)-5-iodobenzoate (I12)

To the mixture of 2-hydroxy-5-iodobenzoic acid (**I11**) (800 mg, 3.03 mmol) in MeOH (16 mL) was added dropwise oxalyl chloride (0.79 mL, 9.09 mmol) at 0°C. The reaction mixture was heated to reflux and stirred for 5 hours. The reaction mixture was concentrated and dried in vacuo to give the product (850 mg, 100%). The compound is previously described in the literature [46]. ^1^H NMR (300 MHz, CDCl_3_) δ 10.71 (s, 1H), 8.13 (d, *J* = 2.2 Hz, 1H), 7.69 (dd, *J* = 8.8, 2.3 Hz, 1H), 6.77 (d, *J* = 8.8 Hz, 1H), 3.96 (s, 3H). To the reaction mixture of methyl 2-hydroxy-5-iodobenzoate (770 mg, 2.77 mmol) and K_2_CO_3_ (765 mg, 5.54 mmol) in DMF (8 mL) was added dropwise BnBr (0.33 mL, 2.77 mmol) at rt. The reaction mixture was heated at 50°C for overnight. After cooling, the reaction mixture was diluted with EtOAc, washed with water, dried over Na_2_SO_4_, and concentrated under reduced pressure. The reaction mixture was purified by chromatography (silica gel, 75/25 v/v petrolether/EtOAc) to give product (0.871 g, 85% yield). ^1^H NMR (400 MHz, CDCl_3_) δ 8.10 (d, *J* = 2.4 Hz, 1H), 7.68 (dd, *J* = 8.7, 2.4 Hz, 1H), 7.49–7.43 (m, 2H), 7.42–7.36 (m, 2H), 7.34–7.28 (m, 1H), 6.78 (d, *J* = 8.8 Hz, 1H), 5.16 (s, 2H), 3.90 (s, 3H). ^13^C NMR (101 MHz, CDCl_3_) δ 165.32, 158.04, 141.99, 140.25, 136.33, 128.73, 128.07, 126.91, 122.99, 116.26, 82.39, 70.82, 52.36. HRMS (ESI) for C_15_H_14_O_3_I [M+H]+, calcd 368.9988, found 368.9993.

#### Methyl 2-(benzyloxy)-5-(phenylsulfonyl)benzoate (I13)

To the solution of methyl 2-(benzyloxy)-5-iodobenzoate (**I12**) (200 mg, 0.54 mmol) and benzenesulfinic acid sodium salt (107 mg, 0.65 mmol) in toluene (3.0 mL) under argon were added Pd_2_(dba)_3_ (15 mg, 0.016 mmol), Xantphos (19 mg, 0.033 mmol), Cs_2_CO_3_ (265 mg, 0.82 mmol), and *n*Bu_4_NCl (181 mg, 0.65 mmol). The mixture was heated at 80°C for 1 h. After cooling, the reaction mixture was diluted with EtOAc, washed with water, dried over Na_2_SO_4_, and concentrated under reduced pressure. The reaction mixture was purified by chromatography (silica gel, 80/20 v/v n-hexane/ EtOAc) to give product (130 mg, 63% yield). ^1^H NMR (400 MHz, CDCl_3_) δ 8.39 (d, *J* = 2.5 Hz, 1H), 7.99 (dd, *J* = 8.9, 2.5 Hz, 1H), 7.96–7.89 (m, 2H), 7.60–7.53 (m, 1H), 7.53–7.47 (m, 2H), 7.47–7.41 (m, 2H), 7.38 (t, *J* = 7.2 Hz, 2H), 7.36–7.28 (m, 1H), 7.08 (d, *J* = 8.9 Hz, 1H), 5.23 (s, 2H), 3.91 (s, 3H). LC-MS: m/z calculated for C_21_H_19_O_5_S [M+ H^+^]: 383; found 383.

#### 2-Hydroxy-5-(phenylsulfonyl)benzoic acid (7j)

To the solution of methyl 2-(benzyloxy)-5-(phenylsulfonyl)benzoate (**I12**) (120 mg, 0.31 mmol) in MeOH (5 ml) was added 10% Pd/C (17 mg). Reaction mixture was hydrogenated (1.5 Bar pressure of H_2_) for 2 h (UPLC control) at rt. Then reaction mixture was filtrated through Celite and evaporated. Crude product was dissolved in THF/H_2_O (1:1, 4 mL) and was added LiOH (70 mg, 0.24 mmol) at rt. After completion of reaction (UPLC control) THF was evaporated and to water was added 5% KHSO_4_, precipitate was filtrated to give product (50 mg, 44%). ^1^H NMR (300 MHz, MeOD) δ 8.43 (d, *J* = 2.4 Hz, 1H), 8.00 (dd, *J* = 8.9, 2.4 Hz, 1H), 7.94 (d, *J* = 7.1 Hz, 2H), 7.69–7.54 (m, 3H), 7.10 (d, *J* = 8.9 Hz, 1H). ^13^C NMR (101 MHz, MeOD) 172.16, 166.96, 143.26, 135.40, 134.53, 133.30, 132.04, 130.67, 128.45, 119.87, 114.58. HRMS (ESI) for C_13_H_11_O_5_S [M+H]+, calcd 279.0327, found 279.0333. Purity by HPLC analysis on Adamas C18: at 210 nm—99.66%; at 254 nm—99.61%

#### General procedure for sulfonylation of anilines I14-17

To the solution of aniline (1 equiv.) and TEA (3 equiv.) in DCM (0.1 M) under argon was added 5-(chlorosulfonyl)-2-hydrohybenzoic acid (1 equiv.) at 0°C. The reaction mixture was stirred for 2–18 hours at rt (controlled by UPLC). After the completion of reaction, the mixture was diluted with EtOAc, washed with 1 M HCl, dried over Na_2_SO_4_, and concentrated under reduced pressure. The reaction mixture was purified by chromatography (C18 silica gel, 95/5–0/100 v/v H_2_O/MeCN) to give product.

#### 2-Hydroxy-5-(N-(3’-methoxy-[1,1’-biphenyl]-3-yl)sulfamoyl)benzoic acid (7p)

Compound was obtained according to general procedure to give product (30 mg, 26%).^1^H NMR (400 MHz, DMSO-*d*_6_) δ 10.30 (s, 1H), 8.20 (d, *J* = 2.5 Hz, 1H), 7.81 (dd, *J* = 8.8, 2.5 Hz, 1H), 7.39–7.29 (m, 4H), 7.11–7.04 (m, 3H), 7.02–6.98 (m, 1H), 6.94 (ddd, *J* = 8.3, 2.6, 1.0 Hz, 1H), 3.80 (s, 3H). ^13^C NMR (101 MHz, DMSO) δ 170.35, 164.00, 159.74, 141.14, 141.12, 138.19, 133.34, 130.14, 129.94, 129.84, 129.73, 122.79, 119.33, 118.90, 118.41, 118.38, 113.72, 113.39, 112.04, 55.13.HRMS (ESI) for C_20_H_18_NO_6_S [M+H]+, calcd 400.0855, found 400.0845. Purity by HPLC analysis on Adamas C18: at 210 nm—93.80%; at 254 nm—93.92%

#### 5-(N-(4-benzylphenyl)sulfamoyl)-2-hydroxybenzoic acid (7q)

Compound was obtained according general procedure to give product (43 mg, 18%). ^1^H NMR (400 MHz, MeOD-*d*_4_) δ 8.20 (d, *J* = 2.4 Hz, 1H), 7.75 (dd, *J* = 8.8, 2.4 Hz, 1H), 7.23 (dd, *J* = 8.1, 6.6 Hz, 2H), 7.17–7.02 (m, 5H), 7.01–6.95 (m, 3H), 3.87 (s, 2H). ^13^C NMR (101 MHz, MeOD) δ 172.37, 166.29, 142.49, 139.73, 136.76, 134.86, 131.66, 131.30, 130.64, 129.80, 129.43, 127.05, 123.14, 119.00, 113.94, 41.98. HRMS (ESI) for C_20_H_18_NO_5_S [M+H]+, calcd 384.0906, found 384.0896. Purity by HPLC analysis on Apollo C18: at 210 nm—95.96%; at 254 nm—97.02%.

#### 2-Hydroxy-5-(N-(4-phenoxyphenyl)sulfamoyl)benzoic acid (7t)

Compound was obtained according general procedure to give product (45 mg, 18%). ^1^H NMR (400 MHz, DMSO-*d*_6_) δ 10.01 (s, 1H), 8.07 (d, *J* = 2.4 Hz, 1H), 7.72 (dd, *J* = 8.8, 2.5 Hz, 1H), 7.38–7.31 (m, 2H), 7.12–7.01 (m, 4H), 6.93–6.86 (m, 4H). ^13^C NMR (101 MHz, DMSO-*d*_6_) δ 170.36, 164.51, 156.99, 153.29, 133.16, 132.98, 130.03, 129.83, 129.07, 123.31, 123.23, 119.74, 118.10, 118.07, 114.27. HRMS (ESI) for C_19_H_14_NO_6_S [M+H]-, calcd 384.0542, found 384.0547. Purity by HPLC analysis on Adamas C18: at 210 nm—89.70%; at 254 nm—89.39%.

#### 5-(N-([1,1’-biphenyl]-3-yl)sulfamoyl)-2-hydroxybenzoic acid (7x)

Compound was obtained according general procedure to give product (40 mg, 17%). ^1^H NMR (400 MHz, MeOD) δ 8.31 (d, *J* = 2.5 Hz, 1H), 7.82 (dd, *J* = 8.8, 2.5 Hz, 1H), 7.52–7.47 (m, 2H), 7.44–7.38 (m, 2H), 7.36–7.28 (m, 4H), 7.08–7.04 (m, 1H), 7.00 (d, *J* = 8.8 Hz, 1H). ^13^C NMR (101 MHz, MeOD-*d*_4_) δ 172.28, 166.39, 143.67, 141.76, 139.45, 134.88, 131.83, 131.29, 130.67, 129.87, 128.65, 127.94, 124.44, 121.03, 120.68, 119.19, 113.95. HRMS (ESI) for C_19_H_14_NO_5_S [M+H]-, calcd 368.0593, found 368.0591. Purity by HPLC analysis on Apollo C18: at 210 nm—94.77%; at 254 nm—94.48%.

### Anilines I14-17

***4-Benzylaniline*** *(****I15****)* and ***4-phenoxyaniline (***I16***)*** are commercially available.

#### 3’-Methoxy-[1,1’-biphenyl]-3-amine (I14)

The compound is previously described in the literature [24]. ^1^H NMR (400 MHz, DMSO-*d*_6_) δ 7.33 (t, *J* = 7.9 Hz, 1H), 7.15–7.04 (m, 3H), 6.90 (d, *J* = 8.1 Hz, 1H), 6.83 (s, 1H), 6.77 (d, *J* = 8.7 Hz, 1H), 6.56 (d, *J* = 8.0 Hz, 1H), 5.13 (brs, 2H), 3.80 (s, 3H).

#### [1,1’-Biphenyl]-3-amine (I17)

The compound is previously described in the literature [25]. ^1^H NMR (400 MHz, CDCl_3_) δ 7.60–7.54 (m, 2H), 7.46–7.39 (m, 2H), 7.37–7.31 (m, 1H), 7.23 (t, *J* = 7.8 Hz, 1H), 7.00 (ddd, *J* = 7.6, 1.8, 1.0 Hz, 1H), 6.92 (dd, *J* = 2.2, 1.7 Hz, 1H), 6.69 (ddd, *J* = 7.9, 2.4, 1.0 Hz, 1H), 3.85 (br s, 2H).

## Supporting information

S1 Text Compound activity data, X-ray crystallography data and NMR spectra. (DOCX)

S1 Data Sequence alignment and homology models. (ZIP)
