# Supplementary material for: Structure-based identification of salicylic acid derivatives as malarial threonyl tRNA-synthetase inhibitors
Source: PLoS One. 2024 Apr 1;19(4):e0296995. doi: 10.1371/journal.pone.0296995 (PMC10984466; doi:10.1371/journal.pone.0296995)
Supplement: S1 Text — (DOCX) [file pone.0296995.s001.docx]

Supporting information for

**Structure-based identification of salicylic acid derivatives as malarial threonyl tRNA-synthetase inhibitors**

*Raitis Bobrovs^1,^*, Jekaterina Bolsakova^1^, Jhon Alex Rodriguez Buitrago^1^, Larisa Varaceva^1^, Marija Skvorcova^1^, Iveta Kanepe^1^, Anastasija Rudnickiha^1^, Emilio Parisini^1,2^, Aigars Jirgensons^1,^**

*^1^ Latvian Institute of Organic Synthesis, Aizkraukles 21, Riga, LV1006, Latvia*

*^2^ Department of Chemistry “G. Ciamician”, University of Bologna, Via Selmi 2, 40126 Bologna, Italy*

Table of Contents

[1 Compound activity data 1](#_Toc153798187)

[2 X-ray crystallography data 15](#_Toc153798188)

[3 ^1^H and ^13^C NMR spectra 16](#_Toc153798189)

[4 References 25](#_Toc153798190)

# Compound activity data

Table SI1. The SAR of adenosine binding subsite substituent. Compound activity is expressed as fraction of PfThrRS inhibited at 100 µM compound concentration. IC_50_ values were determined for compounds that inhibited PfThrRS by more than 50% at 100 μM concentration. Compounds are arranged by activity.

| No | Com ID. | Molecular structure | ThrRS inhibition @100 µM, % | IC_50_, μM |
| --- | --- | --- | --- | --- |
|  | MolPort-000-644-251  MS-3294 |  | 100 | 13.1±1.4 |
| 1 | MolPort-005-489-927 |  | 100 | 31±2 |
| 2 | MolPort-000-645-175 |  | 93 | 31±2 |
| 3 | MolPort-000-639-492 |  | 84 | 32±2 |
| 4 | MolPort-001-727-407 |  | 100 | 32±2 |
| 5 | JB-1928 |  | 74±7 | 36±4 |
| 5 | MolPort-000-628-123 |  | 88 | 46±2 |
| 7 | JB-1811 |  | 80±9 | 46±3 |
| 8 | LVV-457 |  | 67±11 | 55±3 |
| 9 | MolPort-000-644-250 |  | 63 | 67±3 |
| 10 | MolPort-000-628-314 |  | 64 | 76±4 |
| 11 | MolPort-000-639-774 |  | 57 | 84±4 |
| 12 | MolPort-005-461-419 |  | 63 | 83±4 |
| 13 | MolPort-000-645-406 |  | 58 | 89±4 |
| 14 | MolPort-000-645-321 |  | 57 | 90±4 |
| 15 | MolPort-000-487-836 |  | 55 | 92±3 |
| 16 | MolPort-000-630-019 |  | 50±6 | 99 |
| 17 | MolPort-002-363-286 |  | 47±5 | 106 |
| 18 | MolPort-000-630-020 |  | 49±7 | 121 |
| 19 | LVV-521 |  | 47±7 | 121±5 |
| 20 | JB-1707 |  | 47±10 |  |
| 21 | JB-1815 |  | 46±6 |  |
| 22 | LVV-470 |  | 45±13 |  |
| 23 | MolPort-000-639-491 |  | 44±7 |  |
| 24 | MolPort-000-628-101 |  | 41±9 |  |
| 25 | MolPort-000-628-503 |  | 40±6 |  |
| 26 | LVV-501 |  | 39±6 |  |
| 27 | LVV-455 |  | 35±11 |  |
| 28 | JB-1718 |  | 35±9 |  |
| 29 | MolPort-000-644-253 |  | 34±11 |  |
| 30 | MolPort-000-639-493 |  | 33±6 |  |
| 31 | MolPort-005-461-413 |  | 33±7 |  |
| 32 | MolPort-000-644-418 |  | 31±10 |  |
| 33 | MolPort-005-836-906 |  | 31±4 |  |
| 34 | LVV-466 |  | 30±21 |  |
| 35 | MolPort-005-490-725 |  | 30±8 |  |
| 36 | Am-1-6 |  | 28±12 |  |
| 37 | MolPort-000-645-169 |  | 28±8 |  |
| 38 | MolPort-005-461-256 |  | 28±7 |  |
| 39 | MolPort-011-233-641 |  | 26±6 |  |
| 40 | MolPort-005-490-184 |  | 24±5 |  |
| 41 | JB-1757 |  | 23±6 |  |
| 42 | LVV-448 |  | 22±11 |  |
| 43 | MolPort-000-487-837 |  | 22±7 |  |
| 44 | MolPort-005-489-990 |  | 21±5 |  |
| 45 | MolPort-002-363-849 |  | 21±7 |  |
| 46 | JB-1736 |  | 20±6 |  |
| 47 | JB-1817 |  | 20±8 |  |
| 48 | MolPort-005-461-441 |  | 20±12 |  |
| 49 | MolPort-046-534-557 |  | 19±6 |  |
| 50 | JB-1794 |  | 18±7 |  |
| 51 | MolPort-005-707-434 |  | 18±8 |  |
| 52 | MolPort-001-807-055 |  | 17±9 |  |
| 53 | JB-1749 |  | 16±4 |  |
| 54 | MolPort-000-644-252 |  | 16±5 |  |
| 55 | Am-4-1 |  | 15±18 |  |
| 56 | MolPort-000-487-838 |  | 15±7 |  |
| 57 | MolPort-005-321-630 |  | 15±8 |  |
| 58 | MolPort-046-531-659 |  | 14±2 |  |
| 59 | LVV-512 |  | 13±4 |  |
| 60 | LVV-491 |  | 13±4 |  |
| 61 | MolPort-000-654-184 |  | 13±6 |  |
| 62 | MolPort-002-361-564 |  | 13±7 |  |
| 63 | JB-1737 |  | 11±3 |  |
| 64 | JB-1810-1 |  | 11±8 |  |
| 65 | MolPort-046-636-480 |  | 11±7 |  |
| 66 | MolPort-005-328-565 |  | 10±7 |  |
| 67 | MolPort-047-512-573 |  | 10±11 |  |
| 68 | MolPort-005-462-016 |  | 9±4 |  |
| 69 | MolPort-005-818-094 |  | 9±8 |  |
| 70 | Am-6-4 |  | 8±4 |  |
| 71 | MolPort-046-636-408 |  | 8±10 |  |
| 72 | MolPort-046-640-330 |  | 8±9 |  |
| 73 | MolPort-047-482-193 |  | 8±10 |  |
| 74 | LVV-459 |  | 7±1 |  |
| 75 | LVV-465 |  | 7±3 |  |
| 76 | Am-2-3-AF |  | 7±3 |  |
| 77 | MolPort-005-490-683 |  | 7±3 |  |
| 78 | Am-17-1 |  | 6±4 |  |
| 79 | JB-1715 |  | 6±1 |  |
| 80 | JB-1809 |  | 6±7 |  |
| 81 | JB-1714 |  | 5±2 |  |
| 82 | MolPort-046-556-083 |  | 5±9 |  |
| 83 | Am-18-1 |  | 4±7 |  |
| 84 | JB-1805 |  | 4±4 |  |
| 85 | MolPort-045-923-934 |  | 4±9 |  |
| 86 | LVV-475 |  | 3±7 |  |
| 87 | JB-1709 |  | 3±1 |  |
| 88 | LVV-456 |  | 2±1 |  |
| 89 | LVV-520 |  | 2±6 |  |
| 90 | JB-1712 |  | 1±2 |  |
| 91 | JB-1801 |  | 1±2 |  |
| 92 | LVV-451 |  | 0±1 |  |
| 93 | Am-9-1 |  | 0±3 |  |
| 94 | Am-15-1 |  | 0±3 |  |
| 95 | JB-1788 |  | 0±5 |  |
| 96 | LVV-514 |  | -2±6 |  |
| 97 | Am-16-1 |  | -3±4 |  |
| 98 | LVV-489 |  | -3±3 |  |
| 99 | JB-1779 |  | -3±2 |  |
| 100 | MolPort-045-952-383 |  | -3±7 |  |
| 101 | MolPort-045-949-778 |  | -4±8 |  |
| 102 | LVV-483 |  | -5±4 |  |
| 103 | LVV-498 |  | -5±4 |  |
| 104 | Am-11-2 |  | -6±9 |  |
| 105 | JB-1766 |  | -6±2 |  |
| 106 | JB-1769 |  | -7±2 |  |
| 107 | MolPort-046-629-425 |  | -7±6 |  |
| 108 | Am-5-6 |  | -8±5 |  |
| 109 | Am-12-1 |  | -8±5 |  |
| 110 | Am-14-1 |  | -9±3 |  |
| 111 | MolPort-045-916-016 |  | -9±8 |  |
| 112 | MolPort-046-636-407 |  | -9±8 |  |
| 113 | JB-1775 |  | -11±8 |  |
| 114 | MolPort-005-360-459 |  | -20±7 |  |
| 115 | MolPort-045-921-367 |  | -22±6 |  |
| 116 | MolPort-045-922-144 |  | -25±8 |  |
| 117 | MolPort-044-692-961 |  | -28±15 |  |
| 118 | MolPort-005-346-417 |  | -39±2 |  |
| 119 | MolPort-045-922-607 |  | -44±10 |  |

Table SI2. The SAR of arginine binding group. Compound activity is expressed as fraction of PfThrRS inhibited at 100 µM compound concentration. IC_50_ values were determined for compounds that inhibited PfThrRS by more than 50% at 100 μM concentration. Compounds are arranged by activity.

| No | Com ID. | Molecular structure | ThrRS inhibition @100 µM, % | IC_50_, μM |
| --- | --- | --- | --- | --- |
|  | MolPort-000-644-251  MS-3294 |  | 100 | 13.1±1.4 |
| 1 | MolPort-000-628-093 |  | 46±8 |  |
| 2 | MolPort-000-632-025 |  | 42±8 |  |
| 3 | MolPort-005-872-521 |  | 20±6 |  |
| 4 | JB-1810-2 |  | 17±8 |  |
| 5 | LVV-473 |  | 0±6 |  |
| 6 | JB-1769-1 |  | -2±5 |  |
| 7 | JB-1713 |  | n.a |  |
|  |  |  |  |  |
| 8 | JB-1876 |  | 11±10 |  |
| 9 | LVV-563 |  | 10±8 |  |
| 10 | JB-1895 |  | 0±6 |  |

Table SI3. The SAR of zinc ion binding group. Compound activity is expressed as fraction of PfThrRS inhibited at 100 µM compound concentration. IC_50_ values were determined for compounds that inhibited PfThrRS by more than 50% at 100 μM concentration. Compounds are arranged by activity.

| No | Com ID. | Molecular structure | ThrRS inhibition @100 µM, % | IC_50_, μM |
| --- | --- | --- | --- | --- |
|  | MolPort-000-644-251  MS-3294 |  | 100 | 13.1±1.4 |
| 1 | MS-3295 |  | 19±8 |  |
| 2 | MS-3297 |  | 16±7 |  |
| 3 | MS-3299 |  | 16±11 |  |
| 4 | MolPort-008-385-777 |  | 11±7 |  |
| 5 | MolPort-001-890-044 |  | 11±6 |  |
| 6 | MolPort-020-111-835 |  | 7±6 |  |
| 7 | MolPort-020-124-499 |  | 6±5 |  |
| 8 | MolPort-009-352-348 |  | 5±2 |  |
| 9 | MolPort-023-229-111 |  | 5±2 |  |
| 10 | MolPort-000-630-143 |  | 2±4 |  |
| 11 | MolPort-009-578-369 |  | 1±2 |  |
| 12 | MolPort-020-113-459 |  | 0±2 |  |
| 13 | MolPort-005-210-215 |  | 0±3 |  |
| 14 | MolPort-009-343-020 |  | 0±5 |  |
| 15 | MolPort-002-705-434 |  | -1±2 |  |
| 16 | MolPort-005-171-762 |  | -3±3 |  |

# X-ray crystallography data

Table SI4. Diffraction data collection and refinement statistics.

| Wavelength (Å) | 1.00 |
| --- | --- |
| Resolution range (Å) | 79.33–2.08 (2.166–2.129) |
| Space group | P 2_1_ 2_1_ 2_1_ |
| R_merge_ (all I+ & I-) | 0.239 |
| Multiplicity | 11.1 |
| Unit cell (Å, °) | 85.78 110.50 113.97 90 90 90 |
| Total reflections | 525473 |
| Unique reflections | 47446 |
| Completeness (%) | 99.89 (99.83) |
| CC1/2 | 0.994 (0.747) |
| R-work | 0.1900 (0.1932) |
| R-free | 0.2400 (0.2418) |
| Ligands | 2 |
| RMS (bonds) (Å) | 0.0154 |
| RMS (angles) (°) | 2.368 |
| Ramachandran favored (%) | 96.0 |
| Ramachandran allowed (%) | 3.3 |
| Ramachandran outliers (%) | 0.7 |

Statistics for the highest-resolution shell are shown in parentheses.

# ^1^H and ^13^C NMR spectra
